# The genome sequence of the Purple-barred Yellow, *Lythria purpuraria* (Linnaeus, 1758) (Lepidoptera: Geometridae)

**DOI:** 10.12688/wellcomeopenres.27817.1

**Published:** 2026-09-14

**Authors:** Roger Vila, Eric Toro-Delgado, Charlotte J. Wright, Joana I. Meier, Mark L. Blaxter, Wellcome Sanger Institute Tree of Life Management, Samples and Laboratory team, Wellcome Sanger Institute Scientific Operations: Sequencing Operations, Wellcome Sanger Institute Tree of Life Core Informatics team, Tree of Life Core Informatics collective, Project Psyche Community

**Affiliations:** 1 Institut de Biologia Evolutiva, UPF-CSIC, Barcelona, Spain; 2 Tree of Life, Wellcome Sanger Institute, Hinxton, England, UK

**Keywords:** Lythria purpuraria, Purple-barred Yellow, genome sequence, chromosomal, Lepidoptera

## Abstract

We present a genome assembly from a male specimen of *Lythria purpuraria* (Purple-barred Yellow; Arthropoda; Insecta; Lepidoptera; Geometridae). The assembly contains two haplotypes with total lengths of 241.87 megabases and 241.80 megabases. Most of the haplotype 1 assembly (99.57%) was scaffolded into 31 chromosomal pseudomolecules, including the Z sex chromosome. Haplotype 2 was assembled to scaffold level. The mitochondrial genome has also been assembled, with a length of 18.13 kilobases. This work is part of Project Psyche, a collaborative programme generating genomes for European butterflies and moths.

## Species taxonomy

Eukaryota; Opisthokonta; Metazoa; Eumetazoa; Bilateria; Protostomia; Ecdysozoa; Panarthropoda; Arthropoda; Mandibulata; Pancrustacea; Altocrustacea; Allotriocarida; Hexapoda; Insecta; Dicondylia; Pterygota; Neoptera; Eumetabola; Endopterygota; Aparaglossata; Panorpida; Amphiesmenoptera; Lepidoptera; Glossata; Neolepidoptera; Heteroneura; Ditrysia; Obtectomera; Geometroidea; Geometridae; Larentiinae; *Lythria*; *Lythria purpuraria* (Linnaeus, 1758) (NCBI:txid505419)

## Background

The Purple-barred Yellow, *Lythria purpuraria* (Figure 1), is a moth of the family Geometridae, subfamily Sterrhinae. Its forewings have a dark yellow or orange background with two reddish transverse fasciae of variable width, often not reaching the inner margin. The medial fascia is sometimes reduced to a costal spot ( Hausmann & Viidalepp, 2012). The hindwings are a flat yellow or orange, with a reddish margin. Sexual dimorphism is present, as males have pectinate antennae.

*L. purpuraria* has a Palaearctic distribution, extending from Spain and Portugal eastwards to north-western China, mostly south of 56° N ( Hausmann & Viidalepp, 2012). Current occurrence records can be viewed at GBIF and iNaturalist. The species is extinct in the British Isles and rare in many parts of Central Europe ( Hausmann & Viidalepp, 2012). This xerothermophilous species inhabits open areas, usually on sandy substrates such as coastal dunes, pastures on limestone, or steppe-like karst landscapes, at altitudes from sea level up to 1,200 m, depending on latitude. Adults are diurnal and usually bivoltine, flying first in April to June and again in July to August; a third generation may fly until late September in the southern part of its range. The larvae are specialists on *Polygonum aviculare*, although they have also been reported on *Rumex confertus* in the European part of Russia ( Hausmann & Viidalepp, 2012).

The Purple-barred Yellow is very similar to other species in the genus *Lythria*, particularly *L. cruentaria*, with which it has often been confused in the literature. The species can be distinguished from one another because the reddish fasciae of *L. cruentaria* always reach the inner wing margin ( Hausmann & Viidalepp, 2012).

This species has been included in phylogenetic studies of the genus *Lythria* ( Õunap *et al.*, 2009) and the tribe Lythrini ( Õunap *et al.*, 2008). The latter study transferred Lythrini from the subfamily Larentiinae to Sterrhinae. However, relationships within the genus were not fully resolved. *L. purpuraria* has also been included in phylogenetic comparative analyses of wing shape and longevity in Geometridae, which found wing shape to be largely phylogenetically conserved ( Ude *et al.*, 2024), and longevity to be mainly predicted by body size ( Holm *et al.*, 2016).

We present a chromosome-level reference genome for the purple-barred yellow, *Lythria purpuraria*, which will be useful for further comparative studies of geometrids and other Lepidoptera ( Wright *et al.*, 2025), as well as for conservation genomics in regions where this species is rare.

**Figure 1.**
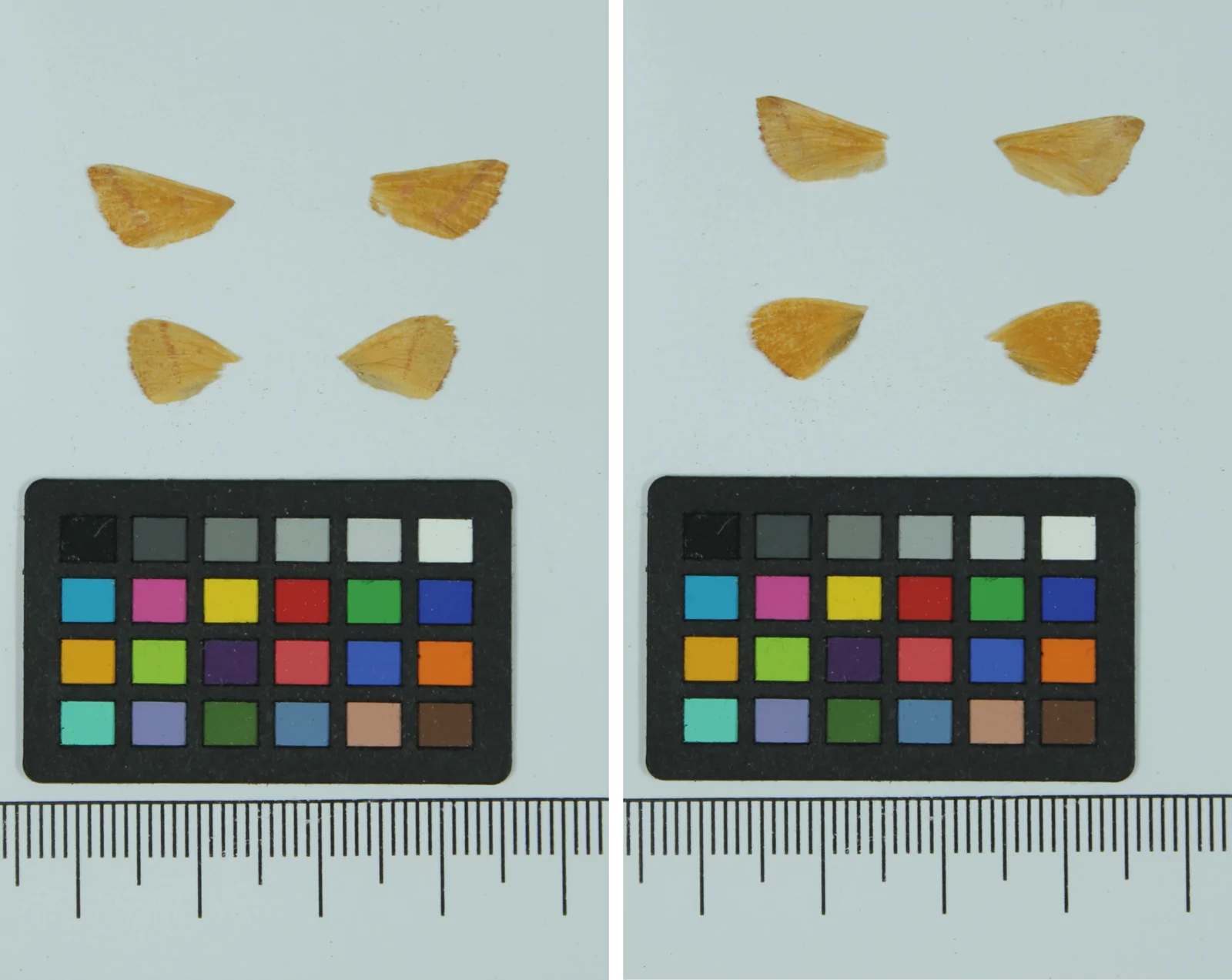
Voucher photographs of the *Lythria purpuraria* specimen (specimen ID SAN28000333, ToLID ilLytPurp1) used for sequencing.

## Methods

### Sample acquisition

The specimen used for genome sequencing was an adult male *Lythria purpuraria* (specimen ID SAN28000333, ToLID ilLytPurp1, Figure 1), collected from Cardedeu, Vallès Oriental, Barcelona, Catalonia, Spain (latitude 41.6365, longitude 2.3442; elevation 186 m) on 2023-08-09. The specimen was collected and identified by Roger Vila (Institut de Biologia Evolutiva).

### Nucleic acid extraction

Protocols for high molecular weight (HMW) DNA extraction developed at the Wellcome Sanger Institute (WSI) Tree of Life Core Laboratory are available on protocols.io ( Howard *et al.*, 2025). For HMW DNA extraction, tissue from the whole organism of ilLytPurp1 was homogenised by powermashing using a PowerMasher II tissue disruptor. HMW DNA was extracted in the WSI Scientific Operations core using the Automated MagAttract v2 protocol. DNA was sheared into an average fragment size of 12–20 kb following the Megaruptor®3 for LI PacBio protocol. Sheared DNA was purified by automated SPRI (solid-phase reversible immobilisation). The concentration of the sheared and purified DNA was assessed using a Nanodrop spectrophotometer and Qubit Fluorometer using the Qubit dsDNA High Sensitivity Assay kit. Fragment size distribution was evaluated by running the sample on the Femto Pulse system. For this sample, the final post-shearing DNA had a Qubit concentration of 16.12 ng/μL and a yield of 757.64 ng, with a fragment size of 13.4 kb. The 260/280 spectrophotometric ratio was 2.3, and the 260/230 ratio was 3.31. The Genomic Quality Number (GQN) was 6.4.

### PacBio HiFi library preparation and sequencing

Library preparation and sequencing took place in the WSI Scientific Operations core. Library PSYCHE15069663 was prepared with the SMRTbell Prep Kit 3.0 (Pacific Biosciences) according to the manufacturer’s instructions. The kit includes reagents for end repair/A-tailing, adapter ligation, post-ligation SMRTbell bead clean-up, and nuclease treatment. Size selection and clean-up were performed using diluted AMPure PB beads (Pacific Biosciences). DNA concentration was quantified with a Qubit Fluorometer v4.0 (Thermo Fisher Scientific) and the Qubit 1X dsDNA HS assay kit. Final library fragment size was assessed with the Agilent Femto Pulse Automated Pulsed Field CE Instrument (Agilent Technologies) using the gDNA 55 kb BAC analysis kit.

The library was sequenced on a Revio instrument (Pacific Biosciences, California, USA). The prepared libraries selected for multiplexing were pooled based on genome size, library molarity and the intended plex level. Primers were annealed and polymerases were bound to generate circularised complexes following the manufacturer’s instructions. Complexes were purified using SMRTbell beads, diluted to the Revio loading concentration, and spiked with a Revio sequencing internal control. The pooled libraries were sequenced on a Revio 25M Plex SMRT cell in a 2-plex run. SMRT Link software (Pacific Biosciences) was used to configure and monitor the run and to perform primary and secondary data analysis.

### Hi-C

***Sample preparation and crosslinking***

Hi-C data were generated from the ilLytPurp1 whole organism sample using the Arima-HiC v2 kit (Arima Genomics). Following the manufacturer’s instructions, tissue was fixed and DNA crosslinked using TC buffer to a final formaldehyde concentration of 2%. The tissue was homogenised using the Diagnocine Power Masher-II. Crosslinked DNA was digested with a restriction enzyme master mix, biotinylated, and ligated. Clean-up was performed with SPRISelect beads before library preparation. DNA concentration was measured with the Qubit Fluorometer (Thermo Fisher Scientific) and Qubit HS Assay Kit. The biotinylation percentage was estimated using the Arima-HiC v2 QC beads.

***Hi-C library preparation and sequencing***

Biotinylated DNA constructs were fragmented using a Covaris E220 sonicator and size-selected to 400–600 bp using SPRISelect beads. DNA was enriched with Arima-HiC v2 kit Enrichment beads. End repair, A-tailing, and adapter ligation were carried out with the NEBNext Ultra II DNA Library Prep Kit (New England Biolabs), following a modified protocol where library preparation occurs while DNA remains bound to the Enrichment beads. Libraries were amplified with KAPA HiFi HotStart mix and a custom Unique Dual Index (UDI) barcode set (Integrated DNA Technologies). Depending on sample concentration and biotinylation percentage determined at the crosslinking stage, libraries were amplified with 10–16 PCR cycles. Post-PCR clean-up was performed with SPRISelect beads. Libraries were quantified using the AccuClear Ultra High Sensitivity dsDNA Standards Assay Kit (Biotium) and a FLUOstar Omega plate reader (BMG Labtech).

Prior to sequencing, libraries were normalised to 10 ng/μL. Normalised libraries were quantified again to create equimolar and/or weighted 2.8 nM pools. Pool concentrations were checked using the Agilent 4200 TapeStation (Agilent) with High Sensitivity D500 reagents before sequencing. Libraries were sequenced on the Illumina NovaSeq X, generating paired-end 150-bp reads.

### Genome assembly

Prior to assembly of the PacBio HiFi reads, a database of *k*-mer counts ( *k* = 31) was generated from the filtered reads using FastK. The *k*-mer frequency distribution was analysed with GenomeScope (version 2.1.0) ( Ranallo-Benavidez *et al.*, 2020), providing fitted point estimates of genome size, heterozygosity and repeat content.

The HiFi reads were assembled using Hifiasm in Hi-C phasing mode ( Cheng *et al.*, 2021, 2022), producing two haplotypes. Hi-C reads ( Rao *et al.* , 2014) were mapped to contigs using bwa-mem2 ( Vasimuddin *et al.*, 2019). Contigs were further scaffolded with Hi-C data in YaHS ( Zhou *et al.*, 2023), using the --break option for handling potential misassemblies. The scaffolded assemblies were evaluated using Gfastats ( Formenti *et al.* , 2022), BUSCO ( Manni *et al.*, 2021) and MerquryFK ( Rhie *et al.*, 2020).

The mitochondrial genome was assembled using MitoHiFi ( Uliano-Silva *et al.* , 2023), which runs MitoFinder ( Allio *et al.*, 2020) and uses these annotations to select the final mitochondrial contig and to ensure the general quality of the sequence, using the mitochondrial genome of *Pasiphila chloerata* (NC_057249.1) as the reference.

### Assembly curation

The assembly was decontaminated using the Assembly Screen for Cobionts and Contaminants ( ASCC) pipeline. The flat files and maps for curation were generated with TreeVal. Manual curation was conducted primarily in PretextView and HiGlass ( Kerpedjiev *et al.* , 2018). Scaffolds were visually inspected and corrected as described by Howe *et al*. (2021). The two haplotype assemblies were combined for curation. Manual corrections included 11 breaks and 14 joins, which reduced the scaffold count by 2.8%. The curation process is described at sanger-tol/curation-resources. A Hi-C contact map of the haplotype 1 assembly was generated with PretextSnapshot.

### Assembly quality assessment

The MerquryFK tool ( Rhie *et al.*, 2020), run in a Singularity container ( Kurtzer *et al.*, 2017), was used to evaluate *k*-mer completeness and assembly quality for both haplotypes using the *k*-mer databases ( *k* = 31) computed prior to genome assembly. The analysis outputs included assembly QV scores and completeness statistics.

The haplotype 1 assembly was assessed using BlobToolKit ( Challis *et al.*, 2020). PacBio reads were mapped to the haplotype 1 assembly to derive sequence coverage, and BUSCO ( Manni *et al.*, 2021) was used to assess gene-set completeness. DIAMOND BLASTp searches of extracted BUSCO proteins and DIAMOND BLASTx searches of assembly sequences against UniProt Reference Proteomes ( Buchfink *et al.*, 2021), followed by BLASTn searches of sequences without DIAMOND BLASTx hits against the NCBI nt database ( Altschul *et al.*, 1990), supported taxonomic assignment. Coverage, sequence composition, taxonomic assignment and BUSCO results were integrated into a BlobDir for interactive inspection and visualisation. The snail and blob plots were rendered locally from this hosted BlobDir using BlobTk v0.8.3. The scaffold-length radial scale function for the snail plot was set to linear ( Challis & Blaxter, 2026). The blob plot used circular markers with area proportional to scaffold length ( Challis *et al.*, 2020).

We used *busco-alg-painter* v0.1.0, a Python reimplementation and extension of *lep_busco_painter*, to paint Merian elements along chromosomes. Merian elements represent the 32 ancestral linkage groups reconstructed for Lepidoptera ( Wright *et al.*, 2024). The BUSCO-to-Merian reference assignments were derived from *lep_busco_painter*. Complete single-copy and duplicated BUSCO genes from the lepidoptera_odb10 results were assigned to Merian elements. Chromosome identifiers and lengths were obtained from NCBI Datasets, and BUSCO positions were plotted on chromosomes drawn to scale.

## Genome sequence report

### Sequence data

PacBio sequencing of the *L. purpuraria* specimen generated 31.36 Gb (gigabases) from 2.92 million reads. We assembled the genome from these reads. GenomeScope analysis using a *p* = 2 model gave a *k*-mer-based haploid genome size estimate of 237.31 Mb, with heterozygosity of 1.64% and repeat content of 20.82%; the full-model fit was 95.17% (Figure 2). The GenomeScope estimates guided expectations for the assembly. The sequencing data provided approximately 127× coverage of the haplotype 1 assembly. Hi-C sequencing produced 102.47 Gb from 339.31 million reads, which were used to scaffold the haplotype 1 assembly. Table 1 summarises the specimen and sequencing details.

**Figure 2.**
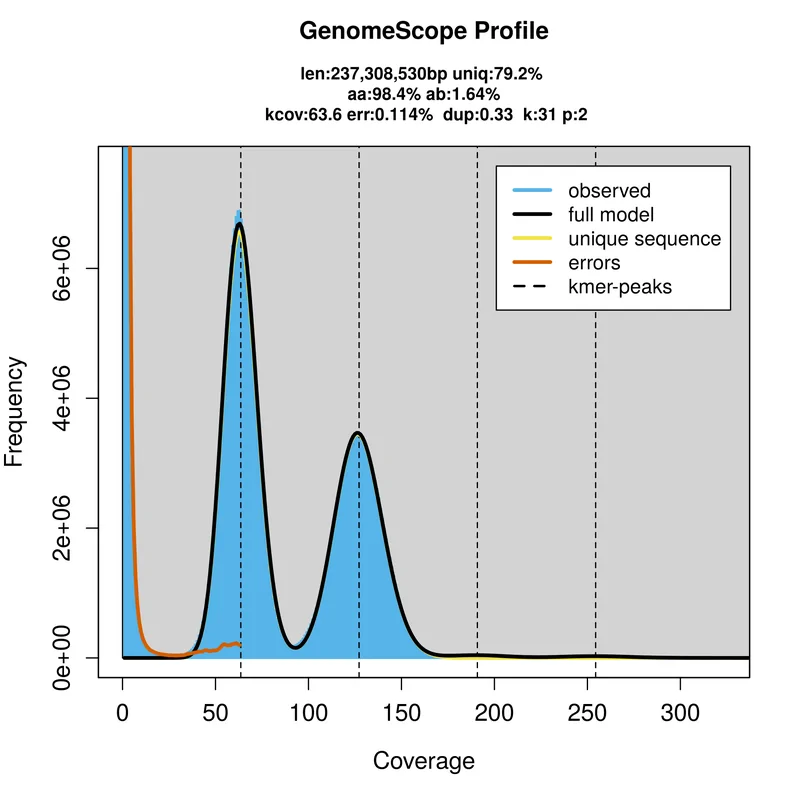
GenomeScope model fitted to the *k*-mer frequency distribution. The plot shows the observed and modelled *k*-mer spectra used to estimate genome size, heterozygosity and repeat content from unassembled sequencing reads.

**Table 1.** Specimen and sequencing data for *L. purpuraria* (BioProject PRJEB82298)

**Platform**	**PacBio HiFi**	**Hi-C**
**ToLID**	ilLytPurp1	ilLytPurp1
**Specimen ID**	SAN28000333	SAN28000333
**BioSample (source individual)**	SAMEA115769829	SAMEA115769829
**BioSample (tissue)**	SAMEA115769879	SAMEA115769879
**Tissue**	whole organism	whole organism
**Instrument**	Revio (1 run)	Illumina NovaSeq X
**Library ID & run accession(s)**	PSYCHE15069663: ERR13946510	ERR13947538
**Read count total**	2.92 million reads	339.31 million read pairs
**Base count total**	31.36 Gb	102.47 Gb

### Assembly statistics

The genome was assembled into two haplotypes using Hi-C phasing. The haplotype 1 assembly was curated to chromosome level, while haplotype 2 was assembled to scaffold level. The haplotype 1 assembly, which is proposed as the reference assembly for this species, has a total length of 241.87 Mb in 69 scaffolds (excluding organelle sequences), with 34 gaps, and a scaffold N50 of 8.73 Mb (Table 2). The haplotype 1 assembly has a scaffold auN of 8.28 Mb.

**Table 2.** Genome assembly statistics for *L. purpuraria*

**Genome assembly**	**Haplotype 1**	**Haplotype 2**
**Assembly name**	ilLytPurp1.hap1.1	ilLytPurp1.hap2.1
**Assembly accession**	GCA_964503375.1	GCA_964503035.1
**Assembly level**	chromosome	scaffold
**Span (Mb)**	241.87	241.80
**Number of chromosomes**	31	-
**Number of contigs**	103	97
**Contig N50 (Mb)**	6.26	6.79
**Number of scaffolds (excluding organelle sequences)**	69	59
**Scaffold N50 (Mb)**	8.73	8.77
**Scaffold auN (Mb)**	8.28	8.30
**Longest scaffold length (Mb)**	11.72	11.73
**Sex chromosomes**	Z	-
**Organelles**	Mitochondrial genome: 18.13 kb	-

Most of the haplotype 1 assembly sequence (99.57%) was assigned to 31 chromosome-level scaffolds, representing 30 autosomes and the Z sex chromosome. These chromosome-level scaffolds, confirmed by Hi-C data, are named according to size (Figure 3; Table 3). Chromosome painting of the haplotype 1 assembly with Merian elements illustrates the distribution of orthologues along chromosomes and highlights patterns of chromosomal evolution relative to Lepidopteran ancestral linkage groups (Figure 4). The Z chromosome was assigned based on the presence of the ancestral Merian element MZ ( Wright *et al.*, 2024).

The mitochondrial genome was also assembled (length 18.13 kb, OZ208040.1). This sequence is included as a contig in the multifasta file of the haplotype 1 genome submission and as a standalone record.

**Figure 3.**
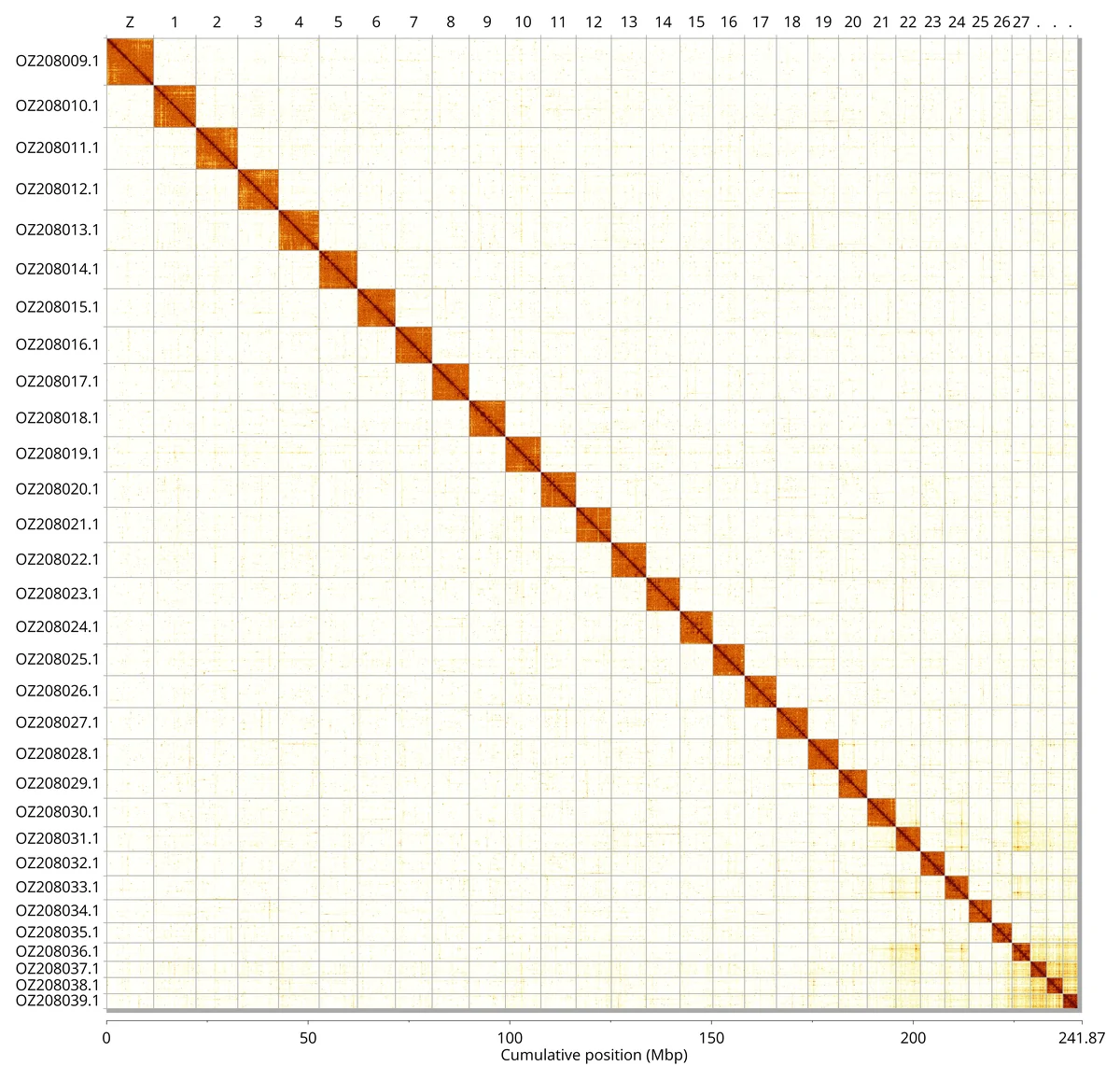
Hi-C contact map of the *L. purpuraria* haplotype 1 assembly. Assembled chromosomes are shown in order of size and labelled along the axes. The plot was generated using PretextSnapshot.

**Table 3.** Chromosomal pseudomolecules in the haplotype 1 genome assembly of *L. purpuraria* ilLytPurp1

**INSDC accession**	**Molecule**	**Length (Mb)**	**GC%**	**Assigned Merian elements**
OZ208010.1	1	10.51	34	M2
OZ208011.1	2	10.34	34	M1
OZ208012.1	3	10.11	34	M3
OZ208013.1	4	10.10	34	M17; M20
OZ208014.1	5	9.48	33	M9
OZ208015.1	6	9.37	33.50	M8
OZ208016.1	7	9.17	33	M12
OZ208017.1	8	9.13	33	M5
OZ208018.1	9	8.97	33	M18
OZ208019.1	10	8.81	33.50	M4
OZ208020.1	11	8.74	33	M16
OZ208021.1	12	8.73	34	M7
OZ208022.1	13	8.70	34	M6
OZ208023.1	14	8.30	33	M21
OZ208024.1	15	8.18	33.50	M15
OZ208025.1	16	7.88	34	M10
OZ208026.1	17	7.88	34	M11
OZ208027.1	18	7.82	33.50	M22
OZ208028.1	19	7.61	34	M13
OZ208029.1	20	7.09	34.50	M23
OZ208030.1	21	7.09	35	M14
OZ208031.1	22	6.09	36	M19
OZ208032.1	23	6.07	34	M24
OZ208033.1	24	5.93	35.50	M26
OZ208034.1	25	5.72	34.50	M28
OZ208035.1	26	4.98	34.50	M27
OZ208036.1	27	4.55	36.50	M25
OZ208037.1	28	4.05	37	M31
OZ208038.1	29	4.02	38	M30
OZ208039.1	30	3.70	36.50	M29
OZ208009.1	Z	11.72	33.50	MZ

**Figure 4.**
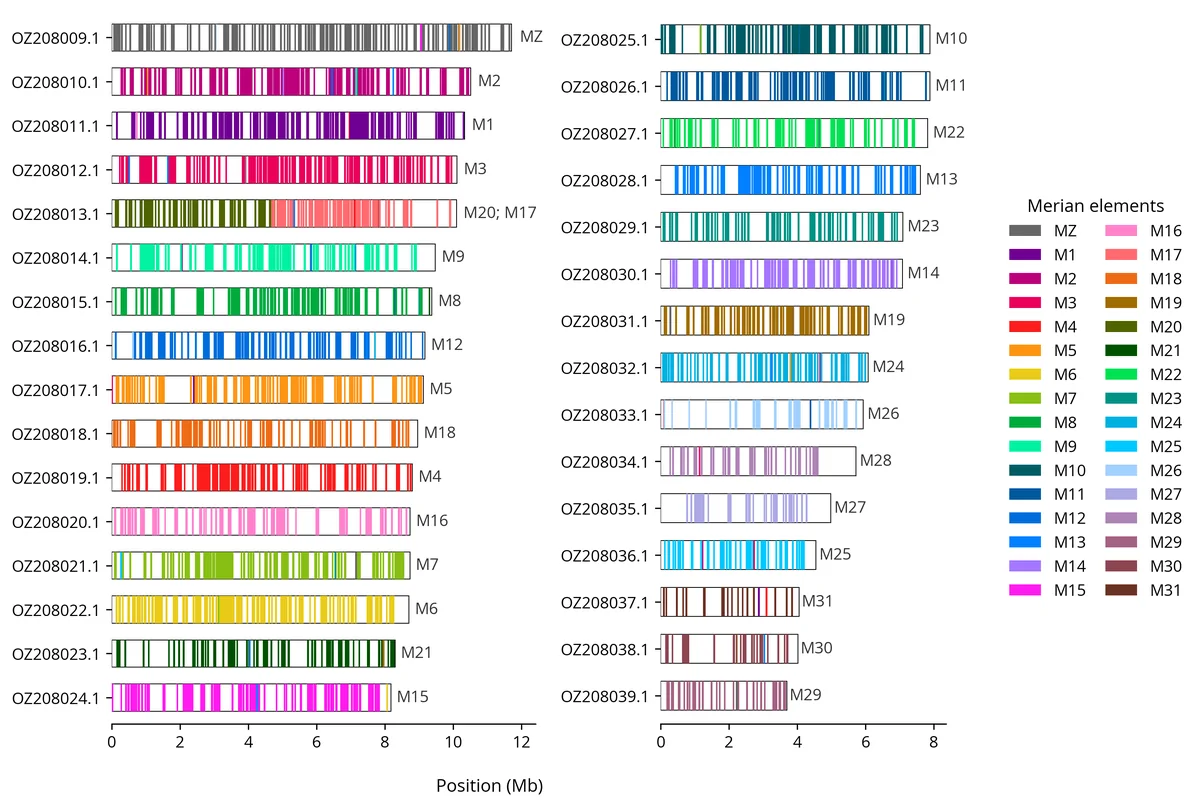
Merian elements painted across chromosomes in the ilLytPurp1.hap1.1 haplotype 1 assembly of *L. purpuraria*. Chromosomes are drawn to scale, with the positions of orthologues shown as coloured bars. Each orthologue is coloured by the Merian element that it belongs to. All orthologues which could be assigned to Merian elements are shown.

### Assembly quality metrics

For the haplotype 1 assembly, the estimated QV is 63.4, and for the haplotype 2 assembly, 63.7. When the two haplotypes are combined, the assembly achieves an estimated QV of 63.6. The *k*-mer completeness is 71.74% for the haplotype 1 assembly, 71.72% for the haplotype 2 assembly, and 99.80% for the combined haplotypes (Figure 5).

**Figure 5.**
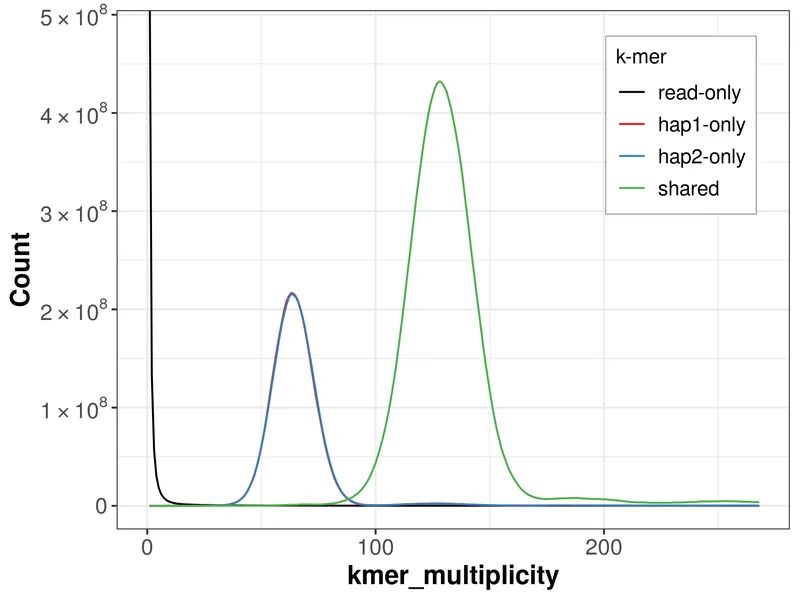
Evaluation of *k*-mer completeness using MerquryFK. This plot illustrates the recovery of *k*‐mers from the original read data in the haplotype 1 assembly, the haplotype 2 assembly and the combined haplotypes. The horizontal axis represents *k*‐mer multiplicity, and the vertical axis shows the number of *k*‐mers. The black curve represents *k*‐mers that appear in the reads but are not assembled. The green curve corresponds to *k*‐mers shared by both haplotypes, and the red and blue curves show *k*‐mers found only in one of the haplotypes.

BUSCO analysis using the lepidoptera_odb10 reference set ( *n* = 5,286) identified 98.5% of the expected gene set (single = 98.0%, duplicated = 0.5%) in the haplotype 1 assembly. For the haplotype 2 assembly, BUSCO analysis identified 98.4% of the expected gene set (single = 98.1%, duplicated = 0.3%).

The snail plot in Figure 6 provides a summary of assembly contiguity, cumulative scaffold count, sequence composition and BUSCO completeness for the haplotype 1 assembly ( Challis & Blaxter, 2026). The relative sizes of the longest scaffold, scaffold N50 and N90, together with the dark-grey scaffold-length distribution, show how much of the assembly is contained in long scaffolds. The outer composition track shows variation in GC, AT and N content, while the central track shows the cumulative scaffold count and the BUSCO inset summarises gene-set completeness.

The blob plot in Figure 7 displays scaffolds from the haplotype 1 assembly by GC proportion and base coverage. Marker area is proportional to scaffold length, and colour indicates taxonomic assignment ( Challis *et al.*, 2020).

**Figure 6.**
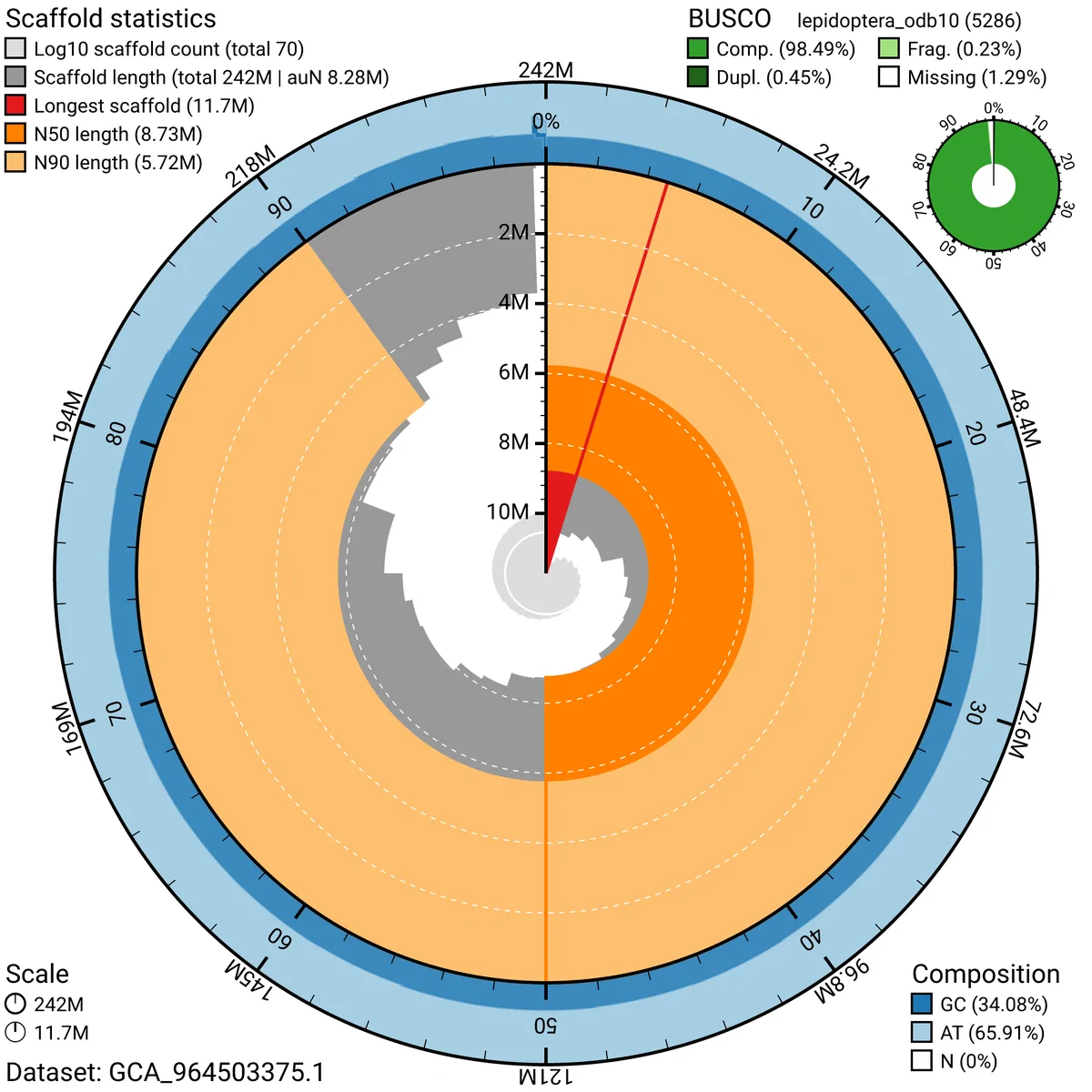
Assembly metrics for ilLytPurp1.hap1.1, the haplotype 1 assembly. The BlobToolKit snail plot provides an overview of assembly metrics and BUSCO gene completeness. The circumference represents the length of the haplotype 1 assembly, and the main plot is divided into 1,000 bins around the circumference. The outermost blue tracks display the distribution of GC, AT, and N percentages across the bins. Scaffolds are arranged clockwise from longest to shortest and are depicted in dark grey. Scaffold length is shown on a linear radial scale, scaled to the longest scaffold. Red shading indicates the longest scaffold, and the deeper orange and pale orange shading represent the N50 and N90 lengths. The summary statistics include scaffold auN, the area under the scaffold Nx curve. The light grey spiral at the centre shows the cumulative scaffold count on a logarithmic scale. A summary of complete, fragmented, duplicated, and missing BUSCO genes in the lepidoptera_odb10 set is presented at the top right. The assembly can be explored interactively in the BlobToolKit viewer.

**Figure 7.**
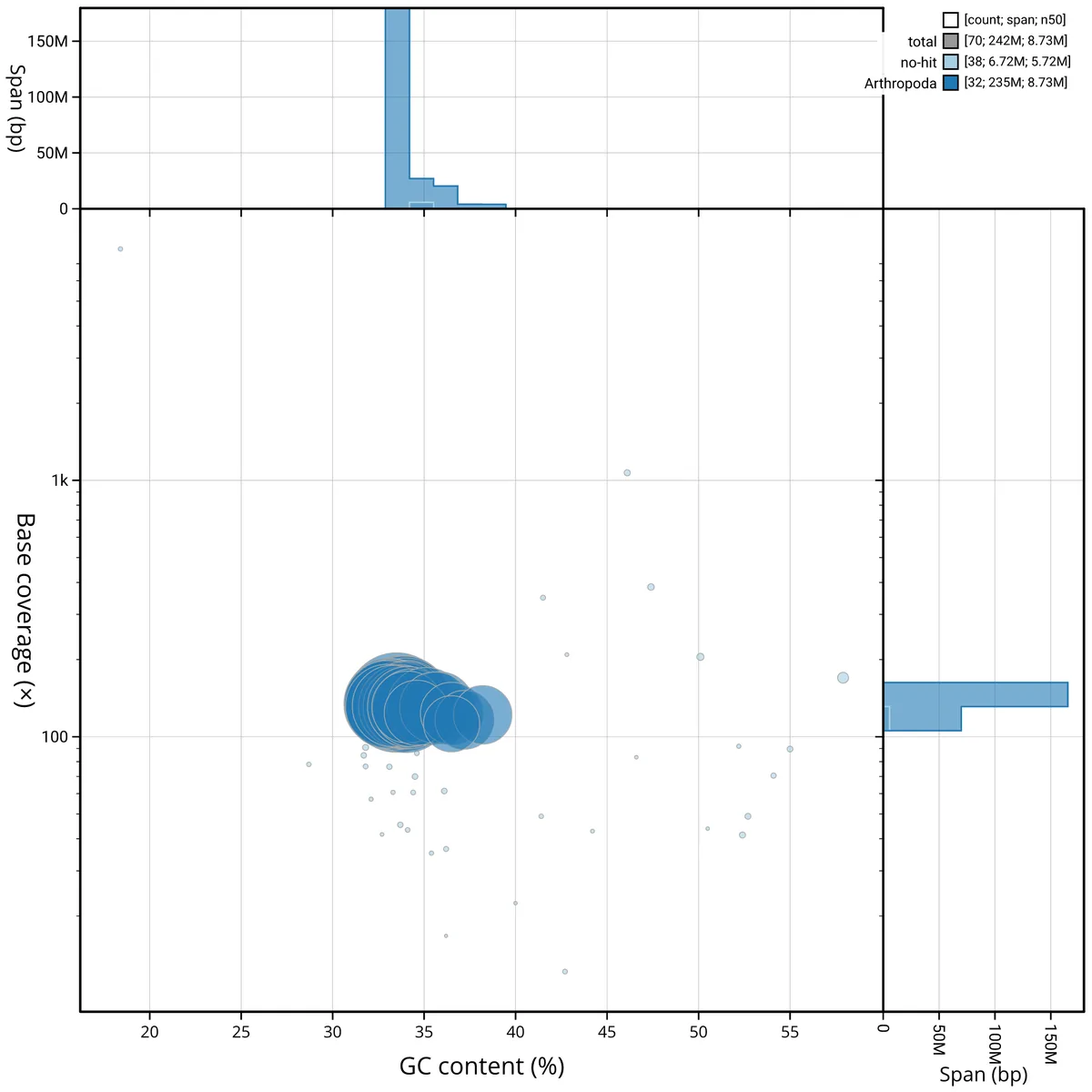
BlobToolKit blob plot for ilLytPurp1.hap1.1, the haplotype 1 assembly. The plot shows base coverage (vertical axis) and GC content (horizontal axis). Circles represent scaffolds; marker area is proportional to scaffold length, and colour indicates taxonomic assignment. The histograms along the axes display the total length of sequences distributed across different levels of coverage and GC content. The assembly can be explored interactively in the BlobToolKit viewer.

Table 4 lists the assembly metric benchmarks adapted from Rhie *et al.*. (2021) the Earth BioGenome Project (EBP) Report on Assembly Standards. The three-part EBP metric, calculated for the haplotype 1 assembly, is **6.C.Q63**.

**Table 4.** Earth BioGenome Project summary metrics for the *L. purpuraria* assembly

**Measure**	**Value**	***Benchmark***
EBP summary (haplotype 1)	6.C.Q63	*6.C.Q40*
Contig N50 length	6.26 Mb	*> 1 Mb*
Scaffold N50 length	8.73 Mb	*= chromosome N50*
Consensus quality (QV)	Haplotype 1: 63.4; haplotype 2: 63.7; combined: 63.6	*≥ 40*
*k*-mer completeness	Haplotype 1: 71.74%; Haplotype 2: 71.72%; combined: 99.80%	*> 90%*
BUSCO	Haplotype 1: C:98.5% [S:98.0%, D:0.5%], F:0.2%, M:1.3%, n:5,286; Haplotype 2: C:98.4% [S:98.1%, D:0.3%], F:0.3%, M:1.3%, n:5,286	*S > 90%; D < 5%*
Percentage of assembly assigned to chromosomes	99.57%	*≥ 90%*

**Notes:** The EBP summary uses log10(Contig N50); chromosome-level (C) or log10(Scaffold N50); Q (Merqury QV). BUSCO v5.7.1 using the lepidoptera_odb10 lineage dataset: C=complete; S=single-copy; D=duplicated; F=fragmented; M=missing; n=orthologues.

### Data availability

European Nucleotide Archive: Lythria purpuraria. Accession number: PRJEB82298; URL: https://identifiers.org/ena.embl/PRJEB82298. The genome sequence is released openly for reuse. The *L. purpuraria* genome sequencing initiative is part of the Sanger Institute Tree of Life Programme ( PRJEB43745) and Project Psyche ( PRJEB71705). All raw sequence data and the haplotype assemblies have been deposited in INSDC databases. The genome will be annotated using available RNA-Seq data and presented through Ensembl at the European Bioinformatics Institute. Raw data and assembly accession identifiers are reported in Table 1 and Table 2.

Pipelines used for genome assembly at the WSI Tree of Life are available at https://pipelines.tol.sanger.ac.uk/pipelines. Table 5 lists software versions used in this study.

**Table 5.** Software versions and sources used for *L. purpuraria*

**Software**	**Version**	**Source**
BLAST	2.14.0	https://ftp.ncbi.nlm.nih.gov/blast/executables/blast+/
BlobToolKit	4.4.5	https://github.com/genomehubs/blobtoolkit
BlobTk (hosted BlobDir)	0.5.1	https://github.com/genomehubs/blobtk
BlobTk (snail and blob plot renderer)	0.8.3	https://github.com/genomehubs/blobtk
BUSCO	5.7.1	https://gitlab.com/ezlab/busco
busco-alg-painter	0.1.0	https://github.com/Karenvn/busco-alg-painter
bwa-mem2	2.2.1	https://github.com/bwa-mem2/bwa-mem2
DIAMOND	2.1.8	https://github.com/bbuchfink/diamond
fasta_windows	0.2.4	https://github.com/tolkit/fasta_windows
FastK	1.2	https://github.com/thegenemyers/FASTK
GenomeScope	2.1.0	https://github.com/tbenavi1/genomescope2.0
Gfastats	1.3.6	https://github.com/vgl-hub/gfastats
Hifiasm	0.19.8-r603	https://github.com/chhylp123/hifiasm
HiGlass	1.13.4	https://github.com/higlass/higlass
MerquryFK	1.1.0-c1	https://github.com/thegenemyers/MERQURY.FK
Minimap2	2.28-r1209	https://github.com/lh3/minimap2
MitoHiFi	3.2.2	https://github.com/marcelauliano/MitoHiFi
MultiQC	1.14; 1.17 and 1.18	https://github.com/MultiQC/MultiQC
Nextflow	24.10.4	https://github.com/nextflow-io/nextflow
PretextSnapshot	0.0.6	https://github.com/sanger-tol/PretextSnapshot
PretextView	1.0.3	https://github.com/sanger-tol/PretextView
samtools	1.21	https://github.com/samtools/samtools
sanger-tol/ascc	0.1.0	https://github.com/sanger-tol/ascc
sanger-tol/blobtoolkit	v0.7.1	https://github.com/sanger-tol/blobtoolkit
sanger-tol/curationpretext	1.4.2	https://github.com/sanger-tol/curationpretext
Seqtk	1.4-r122	https://github.com/lh3/seqtk
Singularity	3.9.0	https://github.com/sylabs/singularity
TreeVal	1.1.1	https://github.com/sanger-tol/treeval
YaHS	1.2a.2	https://github.com/c-zhou/yahs

### Author information

Contributors are listed at the following links:

- Members of the Wellcome Sanger Institute Tree of Life Management, Samples and Laboratory team
- Members of Wellcome Sanger Institute Scientific Operations – Sequencing Operations
- Members of the Wellcome Sanger Institute Tree of Life Core Informatics team
- Members of the Tree of Life Core Informatics collective
- Members of the Project Psyche Community

### Wellcome Sanger Institute – Legal and Governance

The materials that have contributed to this genome note have been supplied by a Tree of Life collaborator. The Wellcome Sanger Institute employs a process whereby due diligence is carried out proportionate to the nature of the materials themselves, and the circumstances under which they have been/are to be collected and provided for use. The purpose of this is to address and mitigate any potential legal and/or ethical implications of receipt and use of the materials as part of the research project, and to ensure that in doing so, we align with best practice wherever possible. The overarching areas of consideration are:

- Ethical review of provenance and sourcing of the material
- Legality of collection, transfer and use (national and international).

Each transfer of samples is undertaken according to a Research Collaboration Agreement or Material Transfer Agreement entered into by the Tree of Life collaborator, Genome Research Limited (operating as the Wellcome Sanger Institute), and in some circumstances, other Tree of Life collaborators.
