# Supplementary material for: The genome sequence of the Purple-barred Yellow, Lythria purpuraria (Linnaeus, 1758) (Lepidoptera: Geometridae)
Source: Wellcome Open Res. 2026 Sep 14;11:662. [Version 1] doi: 10.12688/wellcomeopenres.27817.1 (PMC13694923; doi:10.12688/wellcomeopenres.27817.1)
Supplement: Peer review history

# Reviewer report: Reviewer response for version 1

**DOI:** 10.21956/wellcomeopenres.30610.r174968

**Published:** 2026-10-23

**Authors:** Wai Lok So

**Affiliations:** 1 The Hang Seng University of Hong Kong (Ringgold ID: 272025), Hong Kong, Hong Kong

The manuscript presents a high-quality genome assembly of the Purple-barred Yellow as part of the Project Psyche.

 The background was nicely written, providing with detailed morphological, geographical and biological descriptions on the Purple-barred Yellow. The assembly methods are technically sound, with excellent scaffolding of haplotype 1, including the z sex chromosomes and a complete mitochondrial genome. The BUSCO scores and assembly metrics, particularly for haplotype 1, reaching high sequencing and assembly standards.

 The resulting resource would be very useful for future comparative and conservation genomics research and studies.

 I have no major concerns with the manuscript.

Are the datasets clearly presented in a usable and accessible format, and the assembly and annotation available in an appropriate subject-specific repository?

Yes

Are sufficient details of the sequencing and extraction, software used, and materials provided to allow replication by others?

Yes

Are the rationale for sequencing the genome and the species significance clearly described?

Yes

Are the protocols appropriate and is the work technically sound?

Yes

Reviewer Expertise:

evolution, genomics, arthropods, myriapods

I confirm that I have read this submission and believe that I have an appropriate level of expertise to confirm that it is of an acceptable scientific standard.

# Reviewer report: Reviewer response for version 1

**Published:** 2026-09-14

**Authors:** Automated Benchmarking

**Affiliations:** 1 Wellcome Sanger Institute, Hinxton, UK

As this article is a Genome Note, Automated Benchmarking has been used to determine whether the reported assembly passes three defined checks established by the Earth BioGenome Project: the raw and assembled data are publicly available, the Earth BioGenome Project three-part metric is met, and the BUSCO score passes the stated threshold. For Lythria purpuraria (ilLytPurp1), all three checks passed. Data available: pass. ENA study PRJEB82298, raw-read accessions ERR13946510 and ERR13947538, and assembly accessions GCA_964503375.1 and GCA_964503035.1 are public. Earth BioGenome Project three-part metric: pass. Haplotype 1 is reported as 6.C.Q63, meeting the 6.C.Q40 reference standard. BUSCO: pass. Haplotype 1, which is proposed as the reference assembly for this species, has 98.5% complete BUSCOs (98.0% single-copy and 0.5% duplicated). BUSCO software version: 5.7.1; lineage dataset: lepidoptera_odb10. It meets the target of greater than 90% complete and single-copy BUSCOs. This decision uses values reported in the Genome Note and public accession records.

Reviewer Expertise:

NA

I confirm that I have read this submission and believe that I have an appropriate level of expertise to confirm that it is of an acceptable scientific standard.
